# Overexpression of the ferroptosis-related gene, NFS1, corresponds to gastric cancer growth and tumor immune infiltration

**DOI:** 10.1515/biol-2025-1135

**Published:** 2025-08-08

**Authors:** Zhiyun Mao, Zhongmei Shi, Ming Cui, Xiaohong Ma, Yan Wang, Xiaojie Zhang, Rongrong Jing, Jingchun Wang

**Affiliations:** Department of Laboratory Medicine, Affiliated Hospital of Nantong University, Medical School of Nantong University, No. 20 Xisi Road, Nantong, 226001, China; Department of Laboratory Medicine, The People’s Hospital of Rugao, Rugao, 226500, China; Department of Pathology, Affiliated Hospital of Nantong University, Nantong, 226001, China

**Keywords:** NFS1, ferroptosis, gastric cancer, immune infiltration

## Abstract

The current work has further elucidated the expression and functional implication of cysteine desulfurase (NFS1) in gastric cancer (GC), the prognostic value, and therapeutic target because of the interaction with tumor immune infiltration and ferroptosis. Transcriptomic data from TCGA and GTEX were analyzed to assess mRNA expression and survival correlation with NFS1 among GC patients. A total of 152 GC cases were retrospectively analyzed. The level of NFS1 expression was upregulated in GC tissues compared to non-tumor gastric tissues, which was related to clinical characteristics and poor prognosis. Downregulation of the NFS1 protein in the GC cell line had an adverse effect on the migration, invasion, and proliferation of cells. In addition, NFS1 and immune correlation analysis showed that the level of NFS1 expression was related to a variety of immune cells and characteristics of the immune microenvironment. Based on functional enrichment analysis, NFS1 may have a role in ferroptosis and the tumor microenvironment (TME), such as epithelial–mesenchymal transition control, and the stromal and immunologic responses. NFS1 is a potential diagnostic and prognostic biomarker linked to ferroptosis and the TME, and provides a novel target for cancer treatment and immunotherapy.

## 1 Introduction

Gastric cancer (GC) is still one of the most common and lethal types of cancers worldwide [1]. The burden from this disease is highest in China, accounting for approximately one-half of the new cases worldwide (total = 478,000) [2,3]. Most patients are diagnosed at a late stage and therefore miss the optimal surgical window for disease intervention [4,5]. Consequently, the detection of characteristic biomarkers at an early stage is essential for improving prognosis.

Cysteine desulfurase (NFS1), a ferroptosis suppressor, is the product of a gene located on human chromosome 20q11.22, which helps catalyze the release of sulfur from cysteine to produce an iron–sulfur cluster (ISC) assembly [6,7]. NFS1 is involved in the biosynthesis of ISCs, which are essential in various cellular processes, including maintaining iron homeostasis. Previous research has demonstrated that the ISC enzyme, NFS1, functions as a ferroptosis suppressor involved in ferroptosis and tumor growth [8,9,10,11,12]. Targeting NFS1 to induce ferroptosis has been under investigation as a potential therapeutic strategy in several types of cancers [13].

Because NFS1 can inhibit ferroptosis, targeting NFS1 might enhance treatment efficiency by increasing cancer cell death. Recent studies have shown that NFS1 is highly expressed in GC tissues and is related to a poor prognosis [14]. NFS1 inhibits ferroptosis in GC by upregulating the STAT3 signaling pathway. This inhibition helps GC cells survive and proliferate, making NFS1 a potential target for therapeutic strategies aimed at inducing ferroptosis to combat GC [14]. Knockdown of NFS1 increases the sensitivity of colorectal cancer cells to the ferroptosis inducer, RSL3, and the chemotherapeutic drug, oxaliplatin, enhancing treatment outcomes [15,16]. Previous research has highlighted that high expression of NFS1 is related to poor prognosis [6,17,18,19], which makes NFS1 a potentially good target for early diagnosis and therapeutic intervention in GC.

Few studies have investigated NFS1 in GC patients. The current study was designed to determine the expression of NFS1 in GC tissues, observe the impact of NFS1 downregulation on GC cells, and analyze the relationship with clinical features and the prognostic value.

## 2 Materials and methods

### 2.1 NFS1 mRNA expression analysis

Transcriptomic data, including mRNA and clinical information, were obtained from the Cancer Genome Atlas database (TCGA [https://portal.gdc.cancer.gov]) and the Genotype-Tissue Expression database (GTEX [https://gtexportal.org/home/]). The Kaplan–Meier Plotter (https://kmplot.com/analysis/) was used to determine the relationship between NFS1 mRNA expression and overall survival (OS) in GC patients. mRNA sequencing data from level 3 in the HTSeq-TPM format were converted to the TPM format for further analysis. Statistical analysis and visualization were performed using R (version 4.2.1) and the ggplot2 package.

### 2.2 Tissue microarrays and immunohistochemical analysis

A total of 152 (46 males and 106 females) GC and paired adjacent tissues were obtained from patients who received treatment in the Affiliated Hospital of Nantong University (Nantong, China) between 2005 and 2010 to evaluate NFS1 expression in relation to clinicopathologic features. Patients were divided into two groups (high and low/no expression) based on the status of NFS1 expression. The following information was collected: gender, age, and tumor-node-metastasis (TNM) stage (tumor invasion [T], lymph node involvement [N], and metastasis [M]). Patients were grouped by age (<60 years [*n* = 37] and ≥60 years (*n* = 115). The TNM staging classified the patients into TNM stages I/II (*n* = 55) and III/IV (*n* = 97). Tumor invasion was as follows: Tis/T1/T2 (*n* = 40) and T3/T4 (*n* = 112). Lymph node involvement was categorized as N0 (*n* = 35), N1a (*n* = 31), N1b (*n* = 37), and N2a/b (*n* = 49). Metastasis was classified as M0 (*n* = 118) and M1 (*n* = 34). None of the patients had preoperative radiotherapy, chemotherapy, or immunotherapy.

Clinical data and follow-up information were retrieved from medical records. Tissue specimens of gastric tissues were paraffin-embedded, and tissue specimens were constructed. Immunohistochemical staining was performed to detect NFS1 expression. Tissue specimen chips were dewaxed and dehydrated, and treated with antigen retrieval using 10 mM sodium citrate buffer (pH 6.0) in a microwave. Inhibition of endogenous peroxidase activity was achieved using 3% H_2_O_2_ for 10 min. Sections were incubated at 4°C overnight with primary antibodies to NFS1 (MG815491S, 1:150 dilution; ABmart), followed by incubation with the secondary antibodies. The nuclei were stained with hematoxylin. NFS1 expression was evaluated using a Vectra Automated Quantitative Pathology Imaging System (version 3.0; PerkinElmer, USA). The degree of staining was rated as follows: 0 (no staining), 1 (pale yellow), 2 (brown-yellow), and 3 (tan). The percentage of positively stained cells was multiplied by the intensity score to provide a final immunohistochemistry score ranging from 0 to 300. Three biological and technical replicates were used for each assay.


**Informed consent:** Informed consent has been obtained from all individuals included in this study.
**Ethical approval:** The research related to human use has been complied with all the relevant national regulations, institutional policies and in accordance with the tenets of the Helsinki Declaration, and has been approved by the authors' institutional review board or equivalent committee and has been approved by the Ethics Committee of the Affiliated Hospital of Nantong University (Approval Number: 2022-L025).

### 2.3 Cell culture and NFS1 knockdown

The HGC-27, AGS, MKN-1, and BGC-823 human GC cell lines and the normal gastric epithelial cell line, GES-1, were obtained from the Chinese Academy of Sciences (Shanghai, China). The cells were maintained in RPMI-1640 medium (Corning, Manassas, VA, USA), supplemented with 10% fetal bovine serum (FBS; Gibco, Grand Island, NY, USA), and incubated in an atmosphere of 5% CO_2_ at 37°C.

Cells were transfected with siRNAs targeting NFS1 for NFS1 knockdown (designed and synthesized by GenePharma). The siRNA sequences targeting NFS1 were as follows: si-NFS1-1 (5′-CCCTTACCTAATCAACTACTATG-3′) and si-NFS1-2 (5′-ATCCAACAACATAGCAATTAAGG-3′). GC cells were seeded at 50–60% confluence in 6-well plates before transfection using Lipofectamine 3000 (Thermo Fisher Scientific), according to the manufacturer’s instructions. The cells were subsequently subjected to cell collection for use in western blot analysis, as well as invasion and migration assays.

### 2.4 Cell proliferation and colony formation assays

The Cell Counting Kit-8 (CCK-8) was used to evaluate the number of proliferating viable cells. The transfected cells were seeded into 96-well plates at 1 × 10^3^ cells/well and incubated for the specified length of time. CCK-8 solution (Beyotime, Shanghai, China) was added to each well, and the cells were subsequently incubated at 37°C. Two hours after administering the CCK-8 reagent, every 24 h thereafter, cell vitality was evaluated at 450 nm (OD450). Transfected cells were seeded onto 6-well plates at a density of 400 cells/well in RPMI-1640 medium with 10% FBS for colony formation experiments. After 2 weeks, the cells were rinsed three times with phosphate-buffered saline, fixed in methanol, and stained with 0.1% crystal violet. Visible colonies were manually counted.

### 2.5 Migration and invasion assays

Transwell inserts (8 μm pore size; Millipore, NY, USA) were used for the migration tests. The top chambers of 24-well plates were filled with 100 μl of the transfected cells, which had been resuspended in serum-free RPMI-1640 medium at a density of 2 × 10^5^ cells/ml. The top chambers were pre-coated with diluted Matrigel (BD) for invasion experiments. The bottom chambers were filled with 500 μl/well of RPMI-1640 medium, which included 20% FBS. Cells that had moved or invaded the bottom surface of the membrane were fixed, stained, and photographed following incubation (16 h for invasion and 24 h for migration). Three randomly chosen fields of vision were used to count the cells. Fifty microliters of Matrigel (30 mg/well; BD Biosciences, San Jose, CA, USA) were used to pre-coat the Transwell membranes.

### 2.6 NFS1 functional enrichment analysis

A network of protein–protein interactions (PPIs) was created based on STRING. The following Metascape analyses (https://metascape.org/gp/index.html#/) were performed on co-expressed genes for gene ontology (GO) enrichment: cellular components (CCs), molecular functions (MFs), and biological pathways. The R packages, “ggplot2” and “clusterProfiler,” were utilized for the abovementioned analyses.

### 2.7 Gene set enrichment analysis (GSEA)

Using the Kyoto Encyclopedia of Genes and Genomes (KEGG) gene sets (c2 v7.5.1), Hallmark (v7.5.1), GO (c5 v7.5.1) BPs, CCs, MFs, and pathway enrichment analysis, an effort was made to demonstrate the role of NFS1 expression in biological processes and signaling pathways in GC. The R package “clusterProfiler” was used to perform the statistical analysis and display the results.

### 2.8 Immune infiltration analysis

The ssGSEA method in the GSVA package (v1.46.0) was used to assess Spearman correlations between NFS1 and immune cells for the TCGA datasets. The Tumor Immune Estimation Resource 2 portal (TIMER2 [https://timer.cistrome.org]) provided the purity-adjusted Spearman correlations between NFS1 and tumor-infiltrating immune cells. Correlation scatter plots were created to visualize results. The stromal, immune, and ESTIMATE scores were calculated using SangerBox3.0 and the ESTIMATE package.

### 2.9 Statistical analysis

X-tile software (v3.6.1) was used to determine the cut-off values for low and high NFS1 expression and statistical analysis of OS (Rimm Lab, Yale School of Medicine, New Haven, CT, USA). A *χ* ^2^ test was used to analyze the correlations between clinical parameters and NFS1 expression. Survival curves were plotted using the Kaplan–Meier technique and compared using the log-rank test. The Cox regression method was adopted in both univariate and multivariate analyses to estimate the prognostic value of NFS1 expression. Statistical analyses were performed using the SPSS software (v22.0; IBM SPSS Statistics, IBM Corporation, Armonk, NY, USA). All continuous data are presented as the mean ± SD or mean ± 95% CI. A *P*-value <0.05 was considered statistically significant.

## 3 Results

### 3.1 Identification of NFS1 mRNA expression and the diagnostic significance

**Figure 1:**
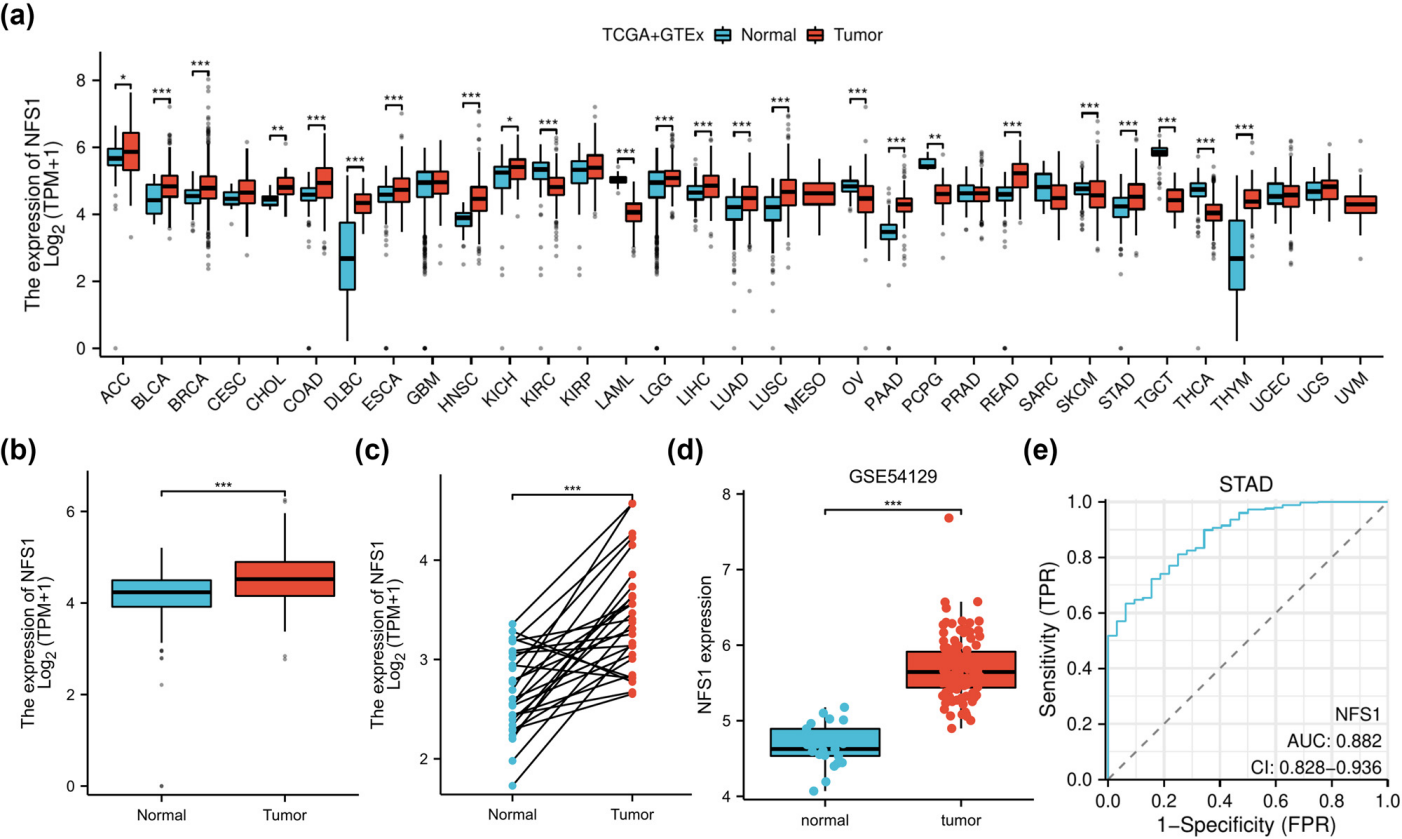
NFS1 mRNA expression in tissues. (a) NFS1 mRNA expression in different tumor types was obtained from the UCSCXenaShiny database. (b) and (c) NFS1 mRNA expression in GC patients and matched adjacent normal samples in The Cancer Genome Atlas databases. (d) NFS1 expression in GC was determined by the GEO dataset. (e) ROC curve analysis for NFS1 expression in GC patients. *P < 0.05.

NFS1 mRNA expression was analyzed in various types of tumors and normal tissues using TCGA and GTEx. NFS1 expression was higher in most types of tumors, including stomach adenocarcinoma (Figure 1a). Specifically, NFS1 mRNA was significantly higher in GC tissue samples compared to matched adjacent normal tissues (Figure 1b and c; *P* < 0.001). Furthermore, in support of these findings, upregulation of NFS1 in GC was confirmed when analyzing the GSE54129 dataset (Figure 1d; *P* < 0.001). Then, receiver operating characteristic (ROC) curve analysis was performed to further determine the diagnostic value of NFS1. The area under the ROC (AUC) was 0.882 (Figure 1e; 95% CI = 0.828–0.936), suggesting that NFS1 has the potential to serve as a diagnostic biomarker in GC.

### 3.2 Correlation of NFS1 protein expression with clinicopathologic features in GC patients

**Figure 2:**
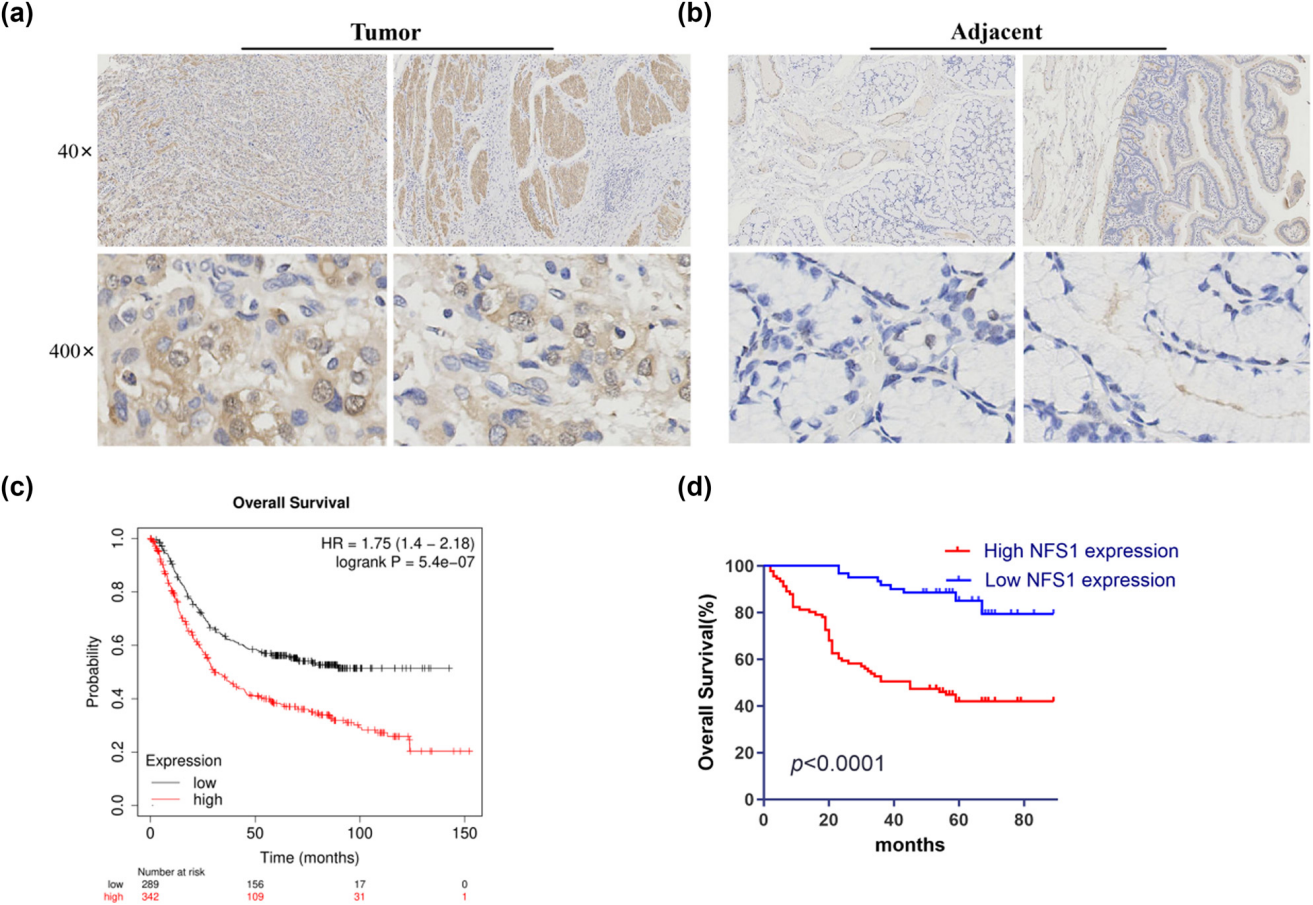
Histochemical analysis and survival data of NFS1 expression in GC tissues. (a) and (b) Histochemical staining results of NFS1 in GC tissues and para-cancerous tissues taken with original 40× magnification (bar = 50 μm) and 400× magnification (bar = 500 μm), respectively. (c) OS curves of NFS1 mRNA expression in GC. (d) High NFS1 protein expression was correlated with a poor prognosis in the clinical GC patient cohort (*n* = 152).

Immunohistochemical labeling was used on a tissue microarray that had 152 pairs of GC and nearby normal tissues because post-transcriptional regulation of mRNA expression is not necessarily indicative of protein levels *in vivo* [20]. Tumor tissues had substantially greater NFS1 protein levels than peritumoral tissues based on mRNA expression patterns (Figure 2a and b). The association between NFS1 expression and clinicopathologic features in patients with GC is shown in Table 1. NFS1 expression and the TNM stage were significantly correlated (*P* = 0.002); patients with advanced stages (III and IV) exhibited greater NFS1 expression. Similarly, because greater expression is more common in patients without metastases (M0), there was a significant correlation between the M stage and NFS1 expression (*P* = 0.033). NFS1 expression, however, did not significantly correlate with age (*P* = 0.953), gender (*P* = 0.599), nerve/vascular invasion (*P* = 0.104), T stage (*P* = 0.138), or N stage (*P* = 0.465). These findings implied that NFS1 expression is impacted by disease severity, especially cancer stage, although other clinical characteristics, including age and gender, do not appear to have a major impact on NFS1 expression.

**Table 1:** Relationship between NFS1 expression and clinicopathological characteristics in GC patients

Characteristic	*n*	Low and no expression	High expression	Pearson *χ* ^2^	*P*
Total	152				
Gender				0.0277	0.599
Male	46	17(37.0)	29(63.0)		
Female	106	44(41.5)	62(58.5)		
Age (years)				0.003	0.953
<60	37	15 (40.5)	22(59.5)		
≥60	115	46(40.0)	69(60.0)		
TNM stage				9.451	0.002
I + II	55	31(56.4)	24(43.6)		
III + IV	97	30(30.9)	67(69.1)		
T				2.2	0.138
Tis + T1 + T2	40	20(50.0)	20(50.0)		
T3, 4b	112	41(36.6)	71(63.4)		
N				2.560	0.465
N0	35	18(51.4)	17(48.6)		
N1a	31	11(35.5)	20(64.5)		
N1b	37	13(35.1)	24(64.9)		
N2a,b	49	19(38.8)	30(61.2)		
M				4.522	0.033
M0	118	42(35.6)	76(64.4)		
M1	34	19(55.9)	15(44.1)		
Nerve/vascular invasion				4.521	0.104
Positive	104	45(43.3)	59(56.7)		
Negative	27	12(44.4)	15(55.6)		
Unknown	21	4(19.0)	17(81.0)		

Tis: Carcinoma *in situ.*

### 3.3 Prognostic potential of NFS1 protein expression in GC

NFS1 expression (HR = 5.546, 95% CI: 2.723–11.293; *P* < 0.001), T stage (HR = 2.142, 95% CI: 1.085–4.230; *P* = 0.028), N stage (HR = 1.329, 95% CI: 1.056–1.673; *P* = 0.015), and M stage (HR = 0.329, 95% CI: 0.141–0.766; *P* = 0.01) were significantly associated with survival (Table 2). Age (HR = 0.824; *P* = 0.499) and gender (HR = 0.931; *P* = 0.797) were not significantly relevant to OS based on univariate analysis (*P* > 0.05; Table 2).

**Table 2:** Univariate analysis of prognostic factors for OS in GC

Factors	Univariate analysis			
	HR	*P*	95% CI	
NFS1 expression	5.546	<0.001	2.723	11.293
High vs low and no				
Age (years)	0.824	0.499	0.469	1.445
<60 vs ≥60				
Gender	0.931	0.797	0.540	1.604
Male vs female				
TNM stage	4.391	<0.001	2.156	8.942
I and II vs III and IV				
T: Tis + T1 + T2 vs T3 + T4	2.142	0.028	1.085	4.230
N: N0 vs N1a vs N1b and N2a + 2b	1.329	0.015	1.056	1.673
M: M0 vs M1	0.329	0.01	0.141	0.766

HR: hazard ratio; CI: confidence interval; Tis: carcinoma *in situ.*

High NFS1 expression represented a powerful independent prognostic factor for poor OS in GC based on multivariate analysis (HR = 4.607, 95% CI: 2.251–9.462; *P* < 0.001; Table 3). Advanced TNM stage (III/IV) was significantly associated with an increased risk of mortality (HR = 3.463, 95% CI: 1.692–7.088; *P* < 0.001; Table 3). The same finding was further verified by the Kaplan–Meier analysis, in which overexpression of NFS1 was significantly related to poor OS in GC patients (Figure 2c and d).

**Table 3:** Multivariate analysis of prognostic factors for OS in GC

Factors	Multivariate analysis			
	HR	*P*	95% CI	
NFS1 expression	4.607	<0.001	2.251	9.462
High vs low and no				
TNM stage	3.463	<0.001	1.692	7.088
I and II vs III and IV				
T: Tis + T1 + T2 vs T3 + T4				
N: N0 vs N1a vs N1b and N2a + 2b				
M: M0 vs M1	0.3362	0.019	0.155	0.848

### 3.4 Upregulated NFS1 expression in GC cell lines

**Figure 3:**
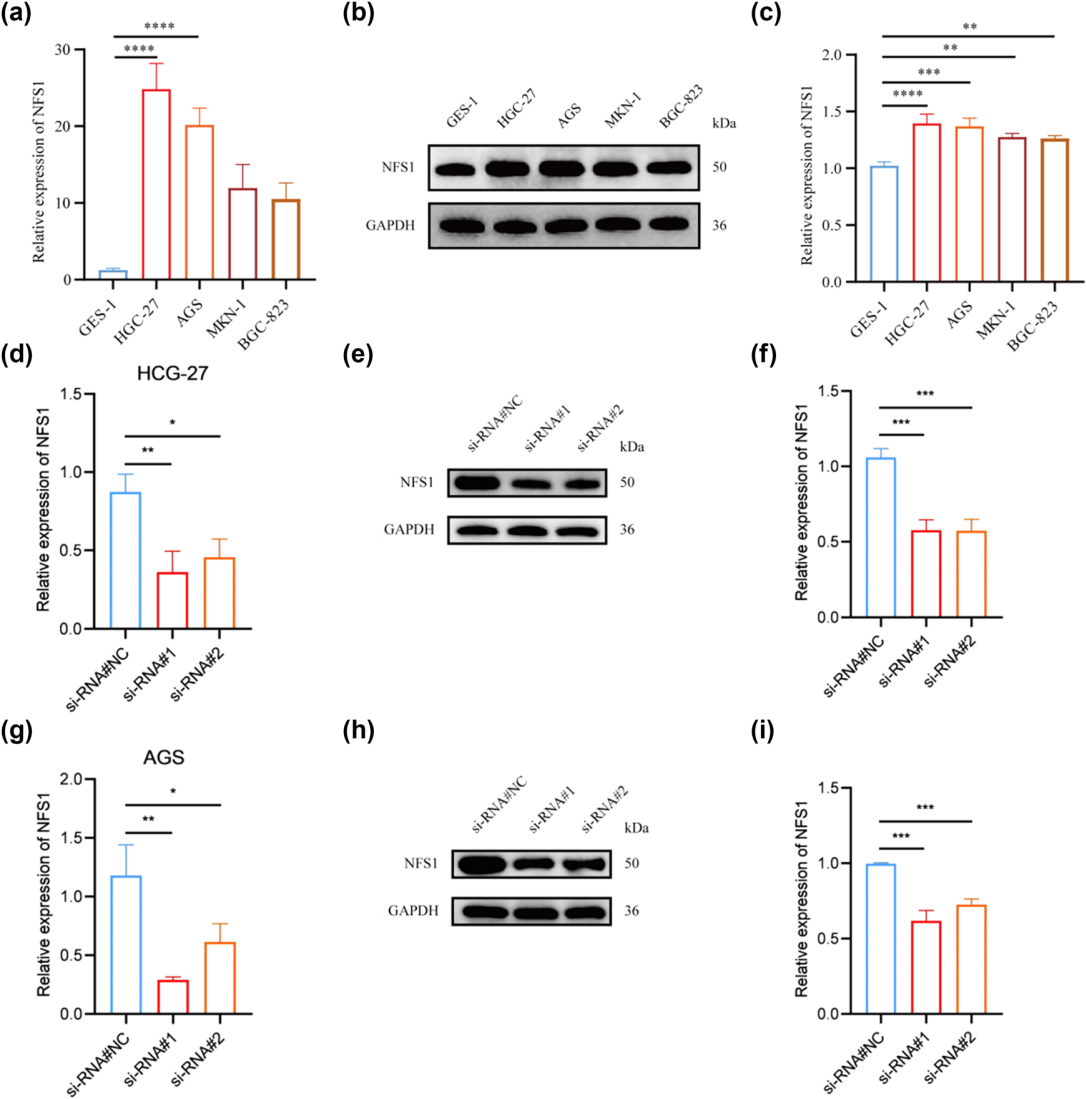
NFS1 expression in GC cell lines. (a) Relative NFS1 expression in GC cell lines (HGC-27, AGS, MKN-1, and BGC-823) compared to a normal gastric epithelial cell line (GES-1). (b) and (c) Western blot showing levels of NFS1 in GC cell lines with GAPDH as a loading control. (d)–(f) qRT-PCR and western blot analysis of knockdown and overexpression efficiency in HGC-27 cells. (g)–(i) qRT-PCR and western blot analysis of knockdown and overexpression efficiency in AGS cells.

NFS1 expression was assessed with a reverse transcription-polymerase chain reaction and western blot analysis in four human GC cell lines (HGC-27, AGS, MKN-1, and BGC-823) and one normal gastric epithelial cell line (GES-1) to determine the phenotypic consequence of NFS1 in GC. NFS1 was highly expressed in GC cell lines compared to GES-1 (Figure 3a–c). Specifically, HGC-27 and AGS had the highest NFS1 levels but the NFS1 levels were low for MKN-1 and BGC-823. Thus, high expression NFS1 and the cell lines with high NFS1 expression (HGC-27 and AGS) were selected. Construction of stable NFS1 knockdown was performed in cells to ascertain knockdown efficacy by qPCR and western blot analysis (Figure 3d–i). The original western blot results are shown in the Supplemental file, WB-NFS1.

### 3.5 NFS1 promoted cell proliferation, migration, and invasion *in vitro*

**Figure 4:**
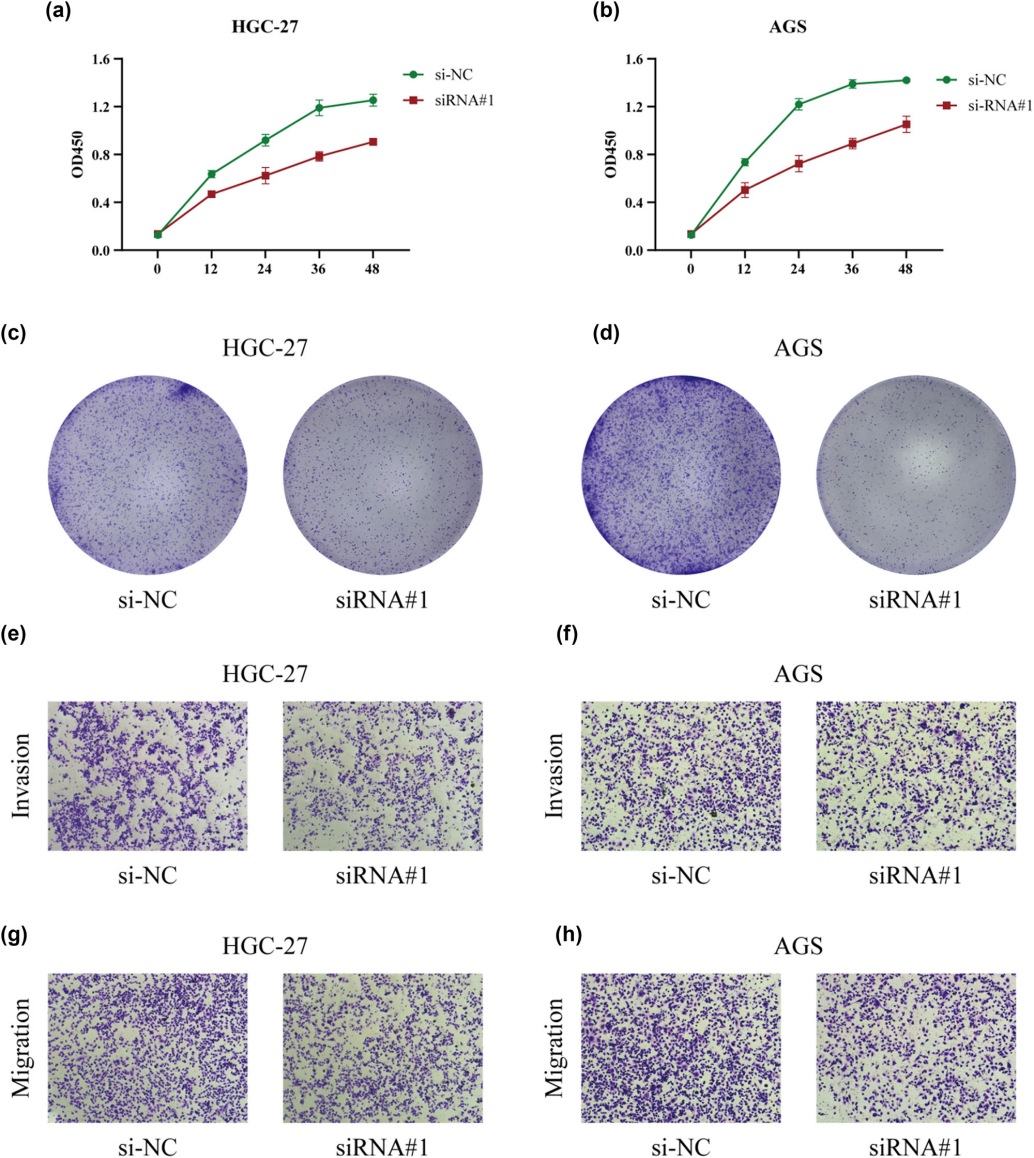
NFS1 promotes proliferation and inhibits apoptosis of GC cells *in vitro*. (a) and (b) CCK-8 assays were used to determine the cell viability of GC cells transfected with si-RNA or si-NC with relative control. (c) and (d) Representative images of colony formation induced by si-NC and si-RNA cells. (e)–(h) Migration and invasion assays were used to investigate the vertical migration and invasion abilities with NFS1 knockdown in GC cells.

Cell viability was measured using CCK-8 and colony-forming assays to determine the role of NFS1 in GC cell proliferation. siRNA-mediated knockdown of NFS1 significantly repressed the growth of HGC-27 and AGS cells compared to the negative control, as shown in Figure 4a and b (*P* < 0.05). Similarly, colony formation was significantly reduced in such cells following NFS1 knockdown (*P* < 0.05; Figure 4c and d). Then, we determined whether NFS1 had a potential role in GC metastasis by using the Transwell assay to assess cell migration and invasion. We demonstrated that NFS1 knockdown significantly reduced the migratory and invasive capabilities of GC cells (Figure 4e–h). These results point to a critical role for NFS1 in GC tumorigenesis and progression.

### 3.6 NFS1 expression related to TME score and immune cell infiltration

**Figure 5:**
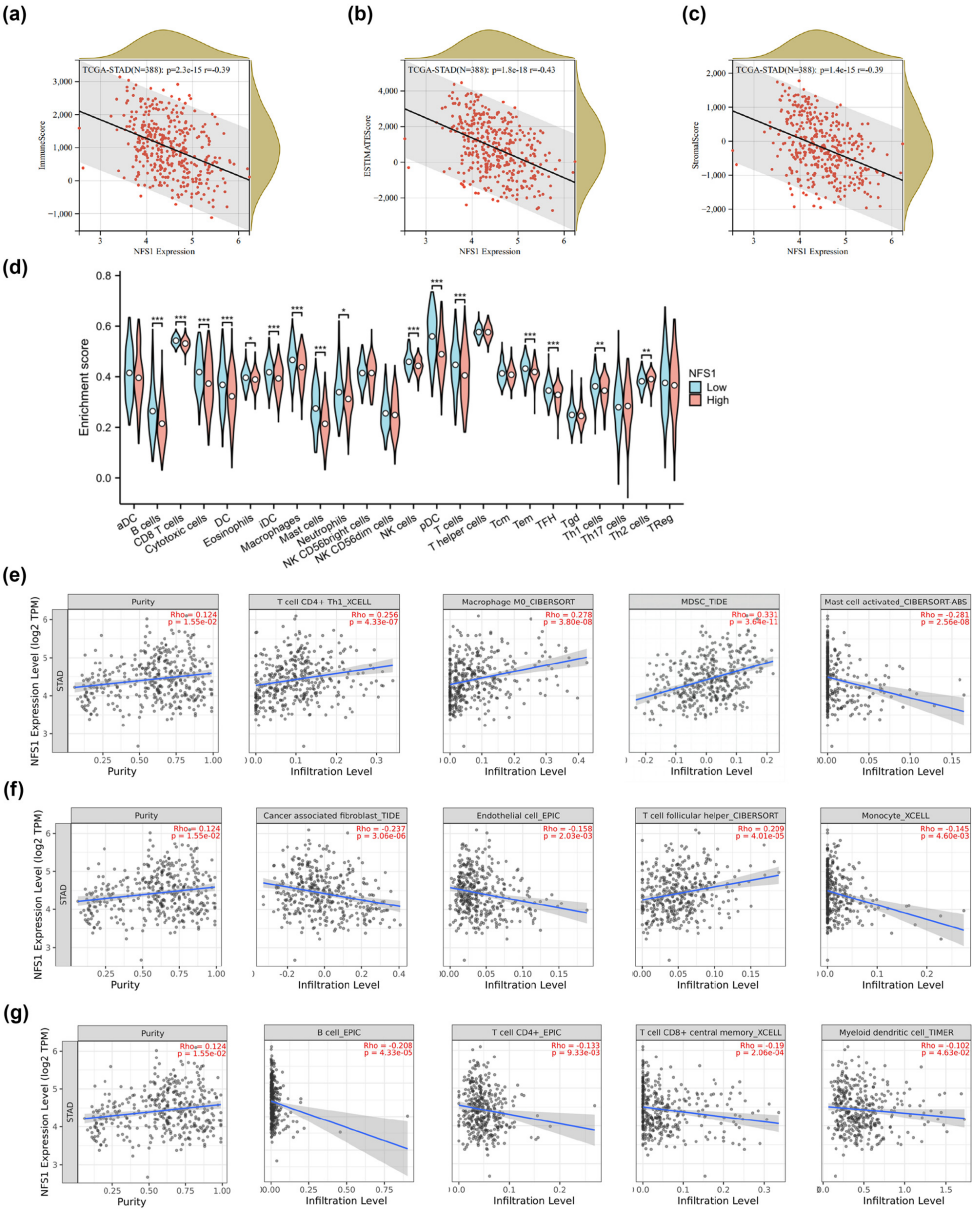
Influence of NFS1 on infiltration of stromal and immune cells. (a)–(c) Relationship between NFS1 expression and immune score, estimated score, and stromal score in GC. (d) Differences of 24 immune cells between the NFS1 high- and low-expression groups. (e)–(g) NFS1 expression associated with 12 kinds of immune cells in TIMER2.0.

The amount and proportion of tumor-infiltrating immune cells are major determinants in patient selection for immunotherapy [21]. The ESTIMATE algorithm was used to calculate the immune, stromal, and estimated scores of GC patients to further elucidate the association between NFS1 expression and tumor microenvironment (TME). The above scores were negatively correlated with NFS1 expression, suggesting that tumor immune activity may be more powerful in patients with lower NFS1 expression, as shown in Figure 5a–c. Moreover, the ssGSEA algorithm was used to investigate the relationship between NFS1 and 24 types of immune cells in GC. NFS1 had a significant correlation with most types of immune cells and may be implicated in the modulation of immune cell infiltration into the TME (Figure 5d).

### 3.7 Correlation between NFS1 expression and tumor-infiltrating immune cells

The TIMER2.0 database was analyzed to provide a deeper understanding of the relationship between the level of NFS1 expression and tumor-infiltrating immune cells, and the tumor purity had a significant positive correlation (*r* = 0.124; *P* = 1.55 × 10^−2^). NFS1 was positively correlated with myeloid-derived suppressor cells (MDSCs) (*r* = 0.331; *P* = 3.64 × 10^−11^; Figure 5e), Th1 cells (*r* = 0.256; *P* = 4.33 × 10^−7^; Figure 5e), and Tfh cells (*r* = 0.209; *P* = 4.01 × 10^−5^; Figure 5f). Stromal cell infiltration, including cancer-associated fibroblasts (CAFs) and endothelial cells, had a negative correlation trend with NFS1 expression but without adequate statistical support (CAFs [*r* = −0.237; *P* = 3.06 × 10^−6^; Figure 5f] and endothelial cells [*r* = −0.158; *P* = 2.03 × 10^−3^; Figure 5e]). In contrast, the negative relevance of NFS1 expression was observed with immune cell types, such as CD8+ T cells (*r* = −0.19; *P* = 2.06 × 10^−4^; Figure 5g), CD4+ T cells (*r* = −0.133; *P* = 9.33 × 10^−3^; Figure 5g), and myeloid dendritic cells (*r* = −0.102; *P* = 4.63 × 10^−2^; Figure 5g). These results suggested that NFS1 expression might influence the tumor immune microenvironment by altering the infiltrating density of immune and stromal cells.

### 3.8 Functional enrichment analysis of NFS1

**Figure 6:**
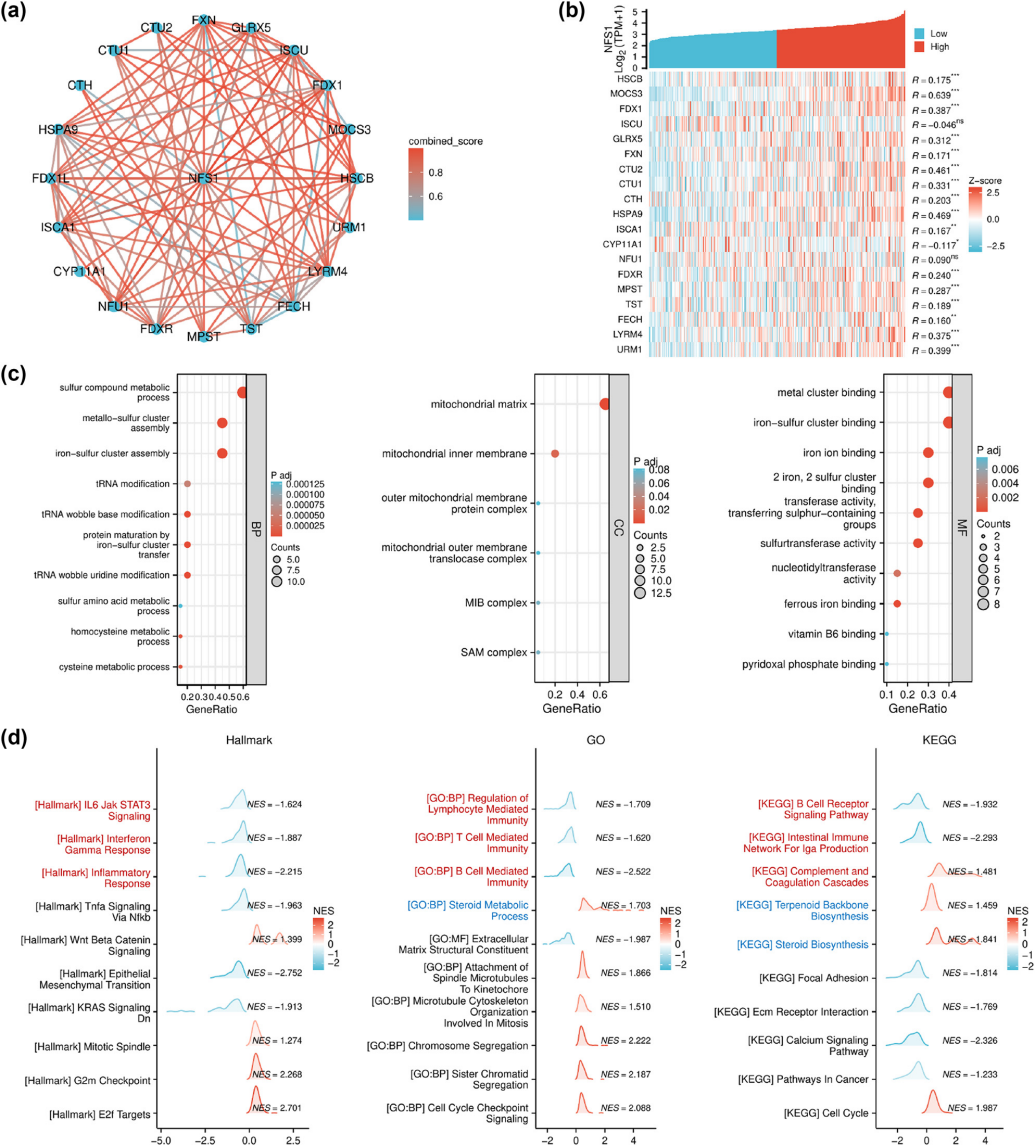
Pathway enrichment analysis of NFS1. (a) PPI network of NFS1 with its 20 interacting proteins. (b) A heatmap of the correlation between NFS1 and the co-expressed genes. ***P < 0.001. (c) GO enrichment analysis of NFS1 and co-expressed genes. (d) Enrichment analysis of the NFS1 GSEA gene in the TCGA database. *P* < 0.05 was considered a meaningful pathway. Red and blue indicate immune- and ferroptosis-related metabolic pathways, respectively.

A functional enrichment analysis was performed to determine the potential functions and pathways related to NFS1 in GC. The STRING database was used to construct the PPI network, and 20 genes interacted with NFS1. The correlations of NFS1 with these genes are represented as a heat map in Figure 6a and b. GO enrichment analysis showed that NFS1 took part in BP, like “ISC assembly,” “metal–sulfur cluster assembly,” “protein maturation by ISC transfer,” “ISC binding,” and “sulfur transferase activity.” The MFs enriched significantly included “iron ion binding.” At the CC level, high NFS1 expression was highly associated with “mitochondrial matrix” and “mitochondrial outer membrane translocase complex” (Figure 6c). All these observations suggest that NFS1 primarily participates in ISC binding and assembly, mitochondrial function, and metabolic processes linked to ferroptosis.

### 3.9 NFS1-related signaling pathways and the possible role in ferroptosis and the TME

We also conducted GSEA to explore the biological processes and signaling pathways associated with NFS1 expression. In hallmark gene sets, NFS1 expression was positively correlated with cell cycle-related pathways, such as “E2F targets,” “G2/M checkpoint,” and “mitotic spindle.” In contrast, NFS1 expression was negatively correlated with KRAS signaling, TGF-α signaling, EMT, and inflammatory responses. The identified DEGs related to NFS1 in GO and KEGG gene sets were involved in the pathway related to the cell cycle checkpoint, signaling that included sister chromatid segregation, cancer pathways, focal adhesion, immune responses, and B- and T-cell-mediated immunity. In addition, a significant positive correlation between NFS1 expression and metabolic processes involving ferroptosis was detected (steroid metabolism, steroid biosynthesis, and terpenoid backbone biosynthesis; Figure 6d). These enrichment results suggested that NFS1 exerts influence on the modulation of GC biological processes through involvement in ferroptosis and TME.

## 4 Discussion

The current study demonstrated significantly upregulated NFS1 expression in GC tissues compared to non-tumor stomach tissues. Downregulation of the NFS1 protein in the GC cell line had a suppressive effect on the migration, invasion, and proliferation of cells. In addition, it was observed that the level of NFS1 expression was related to a variety of immune cells and characteristics of the TME.

In contrast to normal and benign tissues, the level of NFS1 expression in GC tissues was much higher (*χ* ^2^ = 9.451; *P* = 0.002) and the proportion of patients with high expression (*n* = 67 [69.1%]) in patients with TNM stages III + IV was considerably greater than patients with low expression (*n* = 30 [30.9%]). These results suggested that NFS1 may have a role in tumor development [22]. The biological behavior of the tumor likely changes as cancer progresses to a more advanced stage, leading to increased NFS1 expression, which might have a role in tumor migration, resistance, or proliferation [14,15,16,17,18,19]. The level of NFS1 expression was inversely correlated with the tumor stage and OS in GC patients. Knockdown of NFS1 greatly decreased invasion, migration, and proliferation of the GC cell line according to *in vitro* experiments, which is consistent with previous results in other cancer types and indicates the role of NFS1 in carcinogenesis [14,15,16,17,18,19]. These results suggested that during the early and late stages of tumors, NFS1 might serve as an important marker for the development and progression of cancer.

GO enrichment analysis suggested that NFS1 is involved in the biological processes of ISC binding and assembly, cysteine metabolism, and pathways of ferroptosis metabolism. GSEA based on NFS1 expression is related to the cell cycle, cancer, stroma, inflammatory responses, and ferroptosis metabolic pathways. These findings suggested that the potential pathways of NFS1 affecting GC biological processes could be related to ferroptosis and TME-related pathways [6,7,15,16,17].

NFS1 is the key participant in ISC biosynthesis, according to earlier research, and forms ISCs by binding to certain molecular conformations [23]. ISCs have a role in energy conversion, protein translation, and DNA maintenance. When NFS1 is depleted, the iron starvation response is triggered, which causes ferroptosis [6]. Unlike apoptosis, necroptosis, autophagy, pyroptosis, and necrosis, the process-named ferroptosis by Dixon et al. presented a new form of lipid peroxidation-driven regulatory iron-dependent cell death [24]. Ferroptosis has recently gained much attention. Either direct inhibition of lipid peroxidation or iron deprivation prevents iron-dependent phospholipid peroxidation, which is an imbalance between redox homeostasis and cell metabolism [25]. Recent studies have shown that iron death is implicated in various physiologic processes of illness, including organ damage [26], infectious diseases [27], autoimmune disorders [28], and tumorigenesis [29]. Tumor immune activity was higher in patients with low expression of NFS1 than in patients with high expression of NFS1, according to the association between NFS1 expression and immunologic, estimation, and stromal scores in GC. Therefore, tumor cells might develop strategies to inhibit immune function in patients with elevated NFS1 expression. This finding was consistent with previous studies, indicating that NFS1 could be important for immune regulation in the TME [30].

By targeting the OPA3–NFS1 axis, suppression of iron death for protecting cardiac toxicity induced by dox [31,32] and depletion of NFS1 while targeting CAIX enhanced ferroptosis and significantly inhibited tumor growth [12,33]. Based on this study, differential expression of NFS1 in GC tissues may be associated with the ferroptosis pathway. The involvement of NFS1 in the iron metabolism pathway may modulate ISC synthesis, affecting cell sensitivity to ferroptosis. Further investigation is needed to give a more detailed explanation of the mechanism underlying NFS1-regulated ferroptosis in GC.

The TME influences the growth of tumors and is made up of stromal cells, fibroblasts, endothelial cells, immune cells, and various chemokines. The tumor immunotherapy therapeutic response and patient prognosis are determined by the TME [34]. Using the TIMER database, we showed that NFS1 expression had a positive correlation with Th1 cells, Tfh cells, MDSCs, and M0 macrophages but a negative correlation with infiltration of the majority of immune cells, including CD8+ T cells. Numerous investigations have shown that ferroptosis may be one of the ways tumor suppressors partially carry out tumor-suppressive activity. For example, CD8+ T lymphocytes suppress SLC7A11 in tumor cells and secrete IFN-γ to induce ferroptosis [35]. Th1 cells produce IFN-γ, IL-2, and TNF-α, playing a major role in tumor immunity [36]. In liver cancer, the combination of ferroptosis induction and MDSC blockade was shown to be beneficial for effective tumor suppression [37,38]. Additionally, targeting the selenium-GPX4-ferroptosis axis can regulate TFH cell homeostasis, enhancing humoral immunity [38]. These results indicate that NFS1 expression may be an important biomarker for prognosis and is closely related to clinical parameters in GC patients. These findings still need further clinical validation in patients with GC.

Understanding how NFS1 modulates ferroptosis could lead to novel ferroptosis-based therapies, enhancing GC cell sensitivity to this process and improving treatment outcomes [19,39,40]. NFS1 has emerged as an important protein in different cancers and hence represents a promising target for therapeutic intervention. In lung cancer, depletion of NFS1 in combination with cysteine transport inhibition disrupts ISCs, inducing ferroptosis and impairing tumor growth, and may represent a potential therapeutic intervention by targeting NFS1 [41]. High NFS1 expression is associated with poor prognosis, increased tumor aggressiveness, and resistance to ferroptosis in breast cancer, highlighting the role in tumor progression [42]. Similarly, elevated NFS1 expression correlates with poor outcomes in prostate cancer, and targeting NFS1 may enhance the sensitivity of cancer cells to ferroptosis-based therapies [18]. Knockdown of NFS1 increases sensitivity to the ferroptosis inducer, RSL3, and to the chemotherapy drug, oxaliplatin, which leads to better treatment outcomes [43]. These findings showed the broad impact of NFS1 in various cancers. The current study revealed that NFS1 knockdown suppresses GC cell proliferation, migration, and invasion, which is consistent with previous studies [18,19,40,41,42,43]. A significant association was demonstrated between NFS1 expression and TNM and M stages in this study, with higher expression in advanced stages (III and IV) and patients without metastases. These findings suggested that a relationship between NFS1 and tumor progression is required to determine the possible effects of NFS1 in GC patients. The lack of significant correlation with other factors, such as gender, age, and T or N stages, implies that the NFS1 role in the GC population warrants additional study.

The association between NFS1 and the TME was evaluated using the ESTIMATE, stromal, and immune scores to determine the role of NFS1 in the immune microenvironment of GC. The results indicated that NFS1 is negatively correlated with the ESTIMATE, stromal, and immune scores, suggesting that the tumor immune activity of patients with low NFS1 expression was stronger than patients with high NFS1 expression. The ssGSEA algorithm was used to study the relationship between the high and low NFS1 expression groups in the TCGA database and 24 immune cell subsets in GC. More B cells, CD8+ T cells, cytotoxic cells, dendritic cells, eosinophils, immature dendritic cells, macrophages, mast cells, neutrophils, natural killer cells, plasmacytoid dendritic cells, T cells, effector memory T cells, Tfh cells, Th1 cells, and Th2 cells were present in the high NFS1 expression group than in the low NFS1 expression group. The above results indicate that NFS1 may be involved in the TME and affect tumor growth by influencing the infiltration of immune cells. NFS1 appeared to regulate both immune and stromal cell infiltration within TME, with high NFS1 expression associated with lower immune, stromal, and TME scores, suggesting suppressed immune infiltration and poor tumor prognosis. Then, the relationship between NFS1 expression and immune cell infiltration was studied using the TIMER2.0 database. NFS1 was positively correlated with tumor purity (*r* = 0.124; *P* = 1.55 × 10^−2^) and negatively correlated with CD8+ T cells (*r* = −0.19; *P* = 2.06 × 10^−4^), CD4+ T cells (*r* = −0.133; *P* = 9.33 × 10^−3^), myeloid dendritic cells (*r* = −0.102; *P* = 4.63 × 10^−2^), and activated NK cells (*r* = −0.238; *P* = 2.79 × 10^−6^). The results indicated that NFS1 was positively correlated with MDSC (*r* = 0.331; *P* = 3.64 × 10^−11^), Th1 cells (*r* = 0.256; *P* = 4.33 × 10^−7^), and Tfh cells (*r* = 0.209; *P* = 4.01 × 10^−5^). The infiltration of stromal cells was evaluated from the TIMER portal, mainly including CAF and endothelial cells. NFS1 was negatively correlated with CAFs (*r* = −0.237; *P* = 3.06 × 10^−6^) and endothelial cells (*r* = −0.158; *P* = 2.03 × 10^−3^) in GC (Figure 5e–g). Based on the above analysis, the level of NFS1 expression positively correlated with Th1 and Tfh cells, and negatively correlated with CD8+ T cells, mast cells, Th17 cells, B cells, and pDCd. These findings indicated that anti-tumor immunity may have a role in NFS1-mediated carcinogenesis in GC and positioned NFS1 as a potential target of immunotherapy.

The present study provided valuable insight into the relationship between NFS1, immune cell infiltration, and prognosis. However, the present study had some limitations. First, the role of NFS1 in ferroptosis was only inferred from bioinformatics analysis, and the investigation of the underlying mechanisms may be insufficient. Second, immune cell profiling (ssGSEA and TIMER2.0) showed a correlation, but the biological implications remain vague. Further experiments, such as BODIPY-C11 staining, lipid ROS, iron accumulation, and GPX4 levels through *in vitro* ferroptosis assays, are required to illustrate how NFS1 regulates immune and stromal cell infiltration. Third, the current study had a limited sample of GC tissues from a retrospective review of patients. Adding more samples and diversity in further research could affect generalization and the reliability of results, and increasing both would strengthen the power of the study. Testing cytokine profiles or immune checkpoint molecules *in vitro* or from public datasets can be considered in the next step. Fourth, ROC curves and the AUC lacked comparison with standard GC biomarkers. Multivariate ROC curves or combination marker analysis highlighted the diagnostic potential. Finally, clinical validation was lacking in this study, which limits the applicability of the results. Clinical trials to verify the effects in actual patients would give further credence to the conclusions of this research.

## 5 Conclusion

For the first time, the current study showed that NFS1 may function as a possible biomarker for diagnosis and prognosis associated with ferroptosis and TME. NFS1 may also offer a novel target for immunotherapy and medication therapy based on ferroptosis in GC. There were certain restrictions. The direct mechanisms by which NFS1 influences cancer development and prognosis by controlling ferroptosis and TME need to be further studied using more fundamental studies with additional clinical samples.
